# Culture of periprosthetic tissue in blood culture bottles for diagnosing periprosthetic joint infection

**DOI:** 10.1186/s12891-019-2683-0

**Published:** 2019-06-22

**Authors:** Cheng Li, Cristina Ojeda-Thies, Andrej Trampuz

**Affiliations:** 1 Charité – Universitätsmedizin Berlin, Center for Musculoskeletal Surgery, corporate member of Freie Universität Berlin, Humboldt-Universität zu Berlin, and Berlin Institute of Health, Berlin, Germany; 2 Hospital Universitario 12 de Octubre, Madrid, Spain

**Keywords:** Periprosthetic joint infection, Diagnosis, Periprosthetic tissue, Blood culture bottles, Meta-analysis

## Abstract

**Background:**

The purpose of this meta-analysis was to evaluate the diagnostic accuracy of periprosthetic tissue culture in blood culture bottles (BCB) for periprosthetic joint infection (PJI).

**Methods:**

PubMed, Web of Science, and Embase were systematically searched for eligible studies evaluating the diagnostic performance of periprosthetic tissue culture in BCB for the diagnosis of PJI. The pooled data were analysed by Meta-Disc software.

**Results:**

Four studies with a total of 1071 patients were included in this meta-analysis. The summarized estimates showed that periprosthetic tissue culture in BCB may be of great value in PJI diagnosis with a pooled sensitivity of 0.70 (95% confidence interval [CI]; 0.66–0.75), specificity of 0.97 (95% CI: 0.95–0.98); positive likelihood ratio (PLR) of 20.98 (95% CI: 11.52–38.2); negative likelihood ratio (NLR) of 0.28 (95% CI: 0.20–0.40); and diagnostic odds ratio (DOR) of 92.26 (95% CI: 43.93–193.78).

**Conclusions:**

The present meta-analysis showed that periprosthetic tissue in BCB improves the results of microorganism cultures, with a sensitivity of 70% and a specificity of 97%. However, more large-scale, well-performed studies are needed to verify our findings.

## Background

Periprosthetic joint infection (PJI), a severe complication after joint arthroplasty, places a heavy burden on patients and health care resources, including increased mortality, prolonged hospital stays and high medical costs [1]. Although the incidence of periprosthetic hip or knee infection is commonly less than 2% [2, 3]. With the increasing number of arthroplasty procedures, the number of patients who suffer PJI has also increased in relative terms [4]. The diagnosis of PJI remains a challenge, as the yield of conventional microbiological culture methods are less than desired. Thus, using various combinations of diagnostic methods has a supplementary effect and can increase the diagnostic accuracy [5].

Periprosthetic tissue culture is a common method for the diagnosis of PJI, which is included in the diagnostic criteria of the definition of the Infectious Diseases Society of America (IDSA), the Musculoskeletal Infection Society (MSIS) and the European Bone and Joint Infection Society (EBJIS) [6–8]. Previous studies show that the culture method of synovial fluid or sonication fluid in BCB could improve diagnostic sensitivity [9–12]. In recent years, technique of culturing periprosthetic tissue in blood culture bottles (BCB) has been reported. The procedure could be divided into two steps: 1. the intraoperative tissue is placed into the sterile container and transferred to the laboratory; 2. the periprosthetic tissue was homogenized in the biosafety laminar flow hood and inoculated into aerobic and anaerobic BCB [13, 14]. However, it is unknown whether periprosthetic tissue culture in BCB can improve the diagnostic accuracy of PJI. Therefore, we conducted this meta-analysis to evaluate periprosthetic tissue culture in BCB for diagnosing PJI, to provide further evidence for its clinical use.

## Methods

### Search strategy

We searched the electronic databases of PubMed, Web of Science, and Embase for articles published in English until 31 October 2018 using the following medical subject headings (MeSH) or keywords: “periprosthetic joint infection OR prosthetic joint infection OR orthopaedic implant infection” “tissue OR periprosthetic tissue” “blood culture vials OR blood culture bottles OR blood culture system.” The reference lists of the included studies and previous reviews, systematic reviews and meta-analyses were also manually searched to identify potential studies until no additional articles could be found.

### Inclusion criteria

Articles were selected according to the following inclusion criteria: (1) diagnosis of PJI based on a definition, including clinical signs of infection, presence of sinus tract or purulence around the prosthesis, histopathological examination reporting inflammation or significantly positive culture from synovial fluid, periprosthetic tissue samples or sonication fluid [13, 14]; (2) the number of true-positive (TP), true-negative (TN), false-positive (FP), and false-negative (FN) values were clearly reflected as well as computed results of sensitivity and specificity, as the article described; (3) a sample size of more than 15; and (4) published in English.

### Exclusion criteria

Exclusion criteria were as follows: (1) review, meta-analysis, editorials, comments and letters; (2) not related to periprosthetic tissue culture in BCB; and (3) data of diagnostic values are not available or derivable.

### Data extraction

We constructed a data extraction sheet based on the Cochrane Consumers and Communication Review Group data extraction template. The following information was extracted from each study: first author, year of publication, country, enrolment period, number of total cases and infected cases, location, whether to use antibiotics, diagnostic criteria or method, number of tissue samples, antibiotic treatment before sample collection, incubation time, sensitivity and specificity of tissue culture. Furthermore, we contacted the corresponding authors for the missing information, if the above strategy failed. Study quality was assessed according to QUADS-2 guidelines [15].

### Statistical analysis

We used the bivariate random effects regression model to calculate the pooled sensitivity, specificity, PLR, NLR, and DOR [16]. We also established a summary receiver-operating characteristic (SROC) curve calculated under the curve (AUC) and 95% confidence intervals (95% CIs) to adjust for the heterogeneity in positivity criteria [17]. Heterogeneity between these studies was tested using Cochran’s Q test and Higgin’s I-squared statistic, in which I^2^ > 50% or *P* < 0.10 were considered to indicate heterogeneity [18]. Publication bias was assessed using Deeks’ funnel plot. A *P* value less than 0.05 was judged as statistically significant, except where otherwise specified. For the analysis of diagnostic value of periprosthetic tissue BCB, all statistical analyses were performed using Meta-Disc software (version 1.4, Unit of Clinical Biostatistics team, Madrid, Spain).

## Results

### Literature search results

**Fig. 1:**
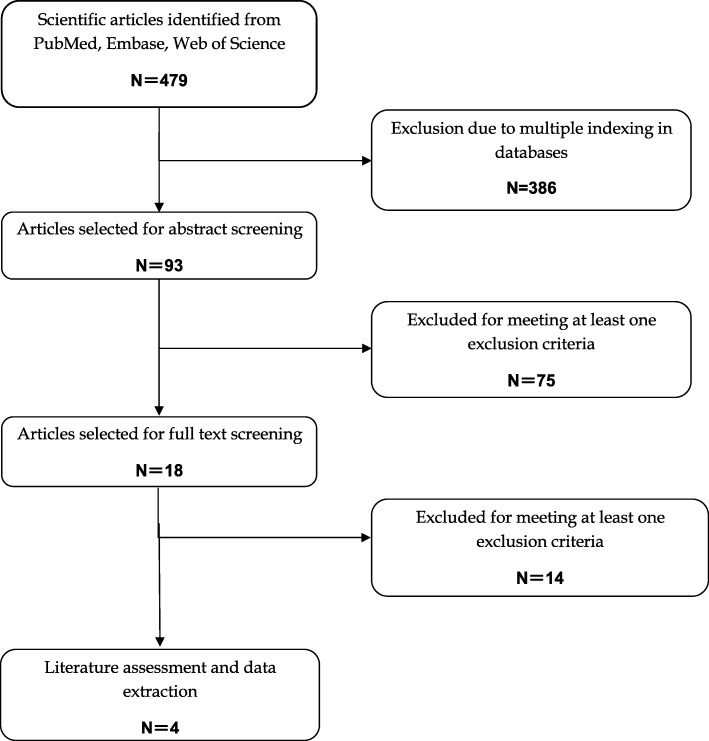
Flowchart of the study inclusion process

The initial search yielded a total of 479 articles, and 386 were excluded because of multiple indexing in different databases. After reviewing the abstract and full article, 75 and 14 were excluded because they were unrelated with the topic studied or did not provide available data, respectively. Finally, 4 articles [1, 13, 14, 19] met the inclusion criteria and were included in this meta-analysis. A flowchart of the study search strategy is shown in Fig. 1.

### Characteristics of eligible studies and quality of the included studies

**Fig. 2:**
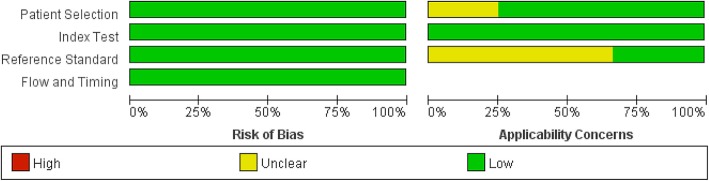
Quality of included studies according to QUADAS-2 guidelines

The primary characteristics of the included studies are presented in Table 1. These studies were published between 2011 and 2018. The sample size ranged from 141 to 369 with a median of 268 patients per study. Among the studies, two were conducted in the USA [1, 13] and two in the UK [14, 19]. Four studies were prospective in design [1, 13, 14, 19]. The prosthetic joint type included hip, knee, shoulder, ankle and elbow arthroplasties. The use of antibiotics prior to surgery was reported in three studies. The assessment of each study was conducted according to the QUADAS-2 guidelines, and results indicated that these included studies were of high quality (Fig. 2).

**Table 1:** Baseline characteristics of patients in included studies

Study	Year	Country	No. of patients	Study design	Prosthetic joint type	Received antibiotics (n, %)	Incubation time	Diagnostic standard
Yan Q [13]	2018	USA	229	Prospective study	Hip/knee/shoulder/elbow	Yes (38, 17%)	Aerobic and anaerobic for 14 days	IDSA
Minassian AM [14]	2014	UK	332	Prospective study	Hip/ankle/shoulder	Yes (NA)	Aerobic and anaerobic for 14 days	P, H, M
Hughes HC [19]	2011	UK	141	Prospective study	Hip/knee	NA	Aerobic and anaerobic for 5 days	H
Peel TN [1]	2016	USA	369	Prospective study	Hip/knee/shoulder/elbow	Yes (60, 16%)	Aerobic and anaerobic for 14 days	IDSA

*Abbreviation: H* histological examination, *IDSA* Infectious Disease Society of America, *M* microbiological or laboratory examination, *NA* not available, *P* presence sinus tract or purulence around the prosthesis

### Diagnostic accuracy of periprosthetic tissue culture in blood culture bottles of periprosthetic joint infection

**Fig. 3:**
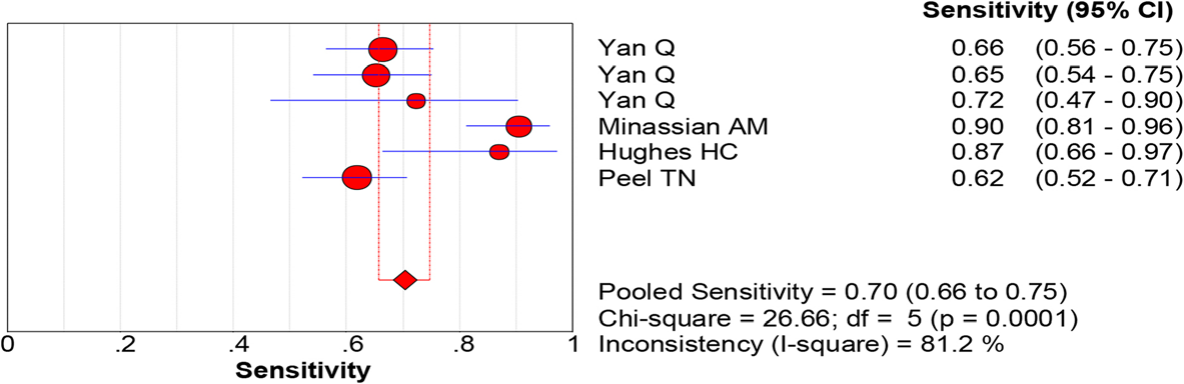
Forest plot of sensitivity for periprosthetic tissue culture in BCB for the diagnosis of PJI

**Fig. 4:**
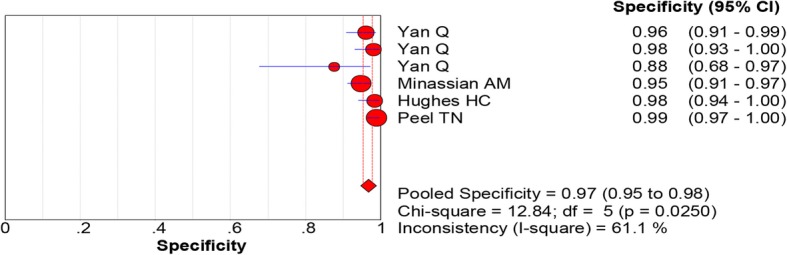
Forest plot of specificity for periprosthetic tissue culture in BCB for the diagnosis of PJI

**Fig. 5:**
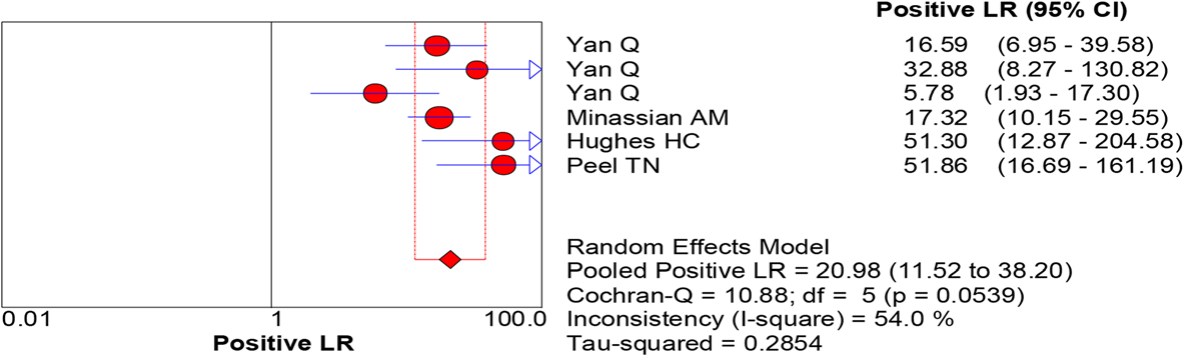
Forest plots of positive likelihood ratio for periprosthetic tissue culture in BCB for the diagnosis of PJI

**Fig. 6:**
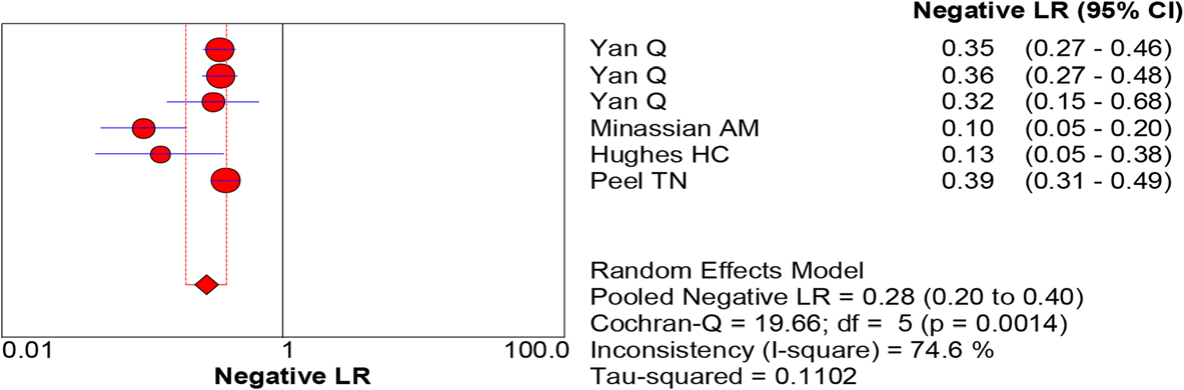
Forest plots of negative likelihood ratio for periprosthetic tissue culture in BCB for the diagnosis of PJI

**Fig. 7:**
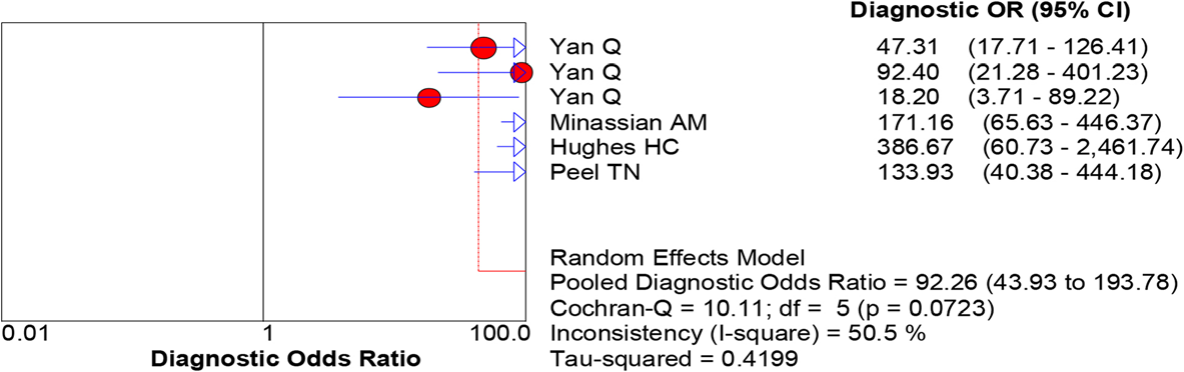
Forest plots of diagnostic odds ratio for periprosthetic tissue culture in BCB for the diagnosis of PJI

**Fig. 8:**
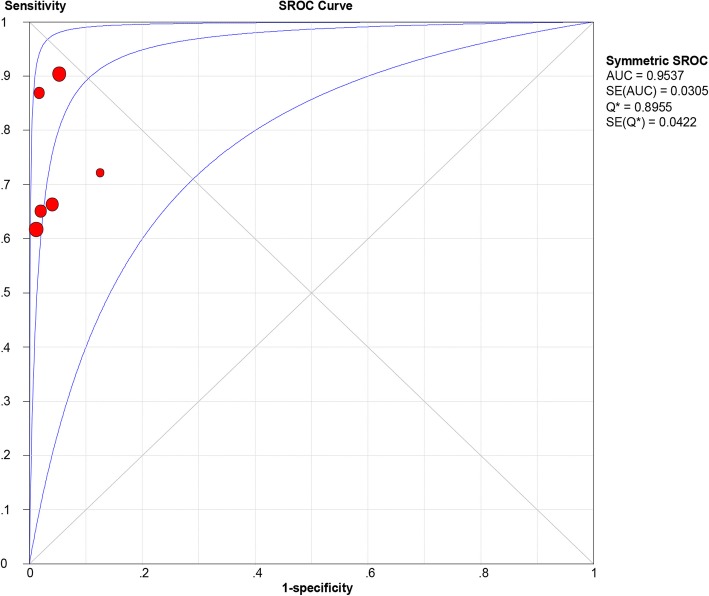
Summary receiver-operating characteristic (SROC) curve for periprosthetic tissue culture in BCB for the diagnosis of PJI. Red circles represent individual studies (see Figs. 3, 4, 5, 6, 7)

All of the included studies reported the data of diagnostic accuracy of periprosthetic tissue culture in BCB. Because the test of heterogeneity was significant for sensitivity (*I*^2^ = 81.2%) and specificity (I^2^ = 61.1%), a random-effects model was used. Pooled results showed that the estimates of sensitivity, specificity, PLR, NLR, and DOR for the detection of PJI using periprosthetic tissue culture in BCB were 0.70 (95% CI: 0.66–0.75), 0.97 (95% CI: 0.95–0.98), 20.98 (95% CI: 11.52–38.20), 0.28 (95% CI: 0.20–0.40), and 92.26 (95% CI: 43.93–193.78), respectively (Figs. 3, 4, 5, 6 and 7). The SROC plot showed the sensitivity and specificity, as well as the 95% confidence intervals and prediction regions, with an AUC of 0.9537 (standard error, 0.0305) (Fig. 8).

### Publication bias

Because the number of included studies was less than 10, we did not assess for publication bias.

## Discussion

In the present meta-analysis, four studies were pooled to evaluate the diagnostic value of periprosthetic tissue in BCB for PJI to provide further evidence for its clinical use. Among the included studies, sensitivity ranged from 0.66 to 0.75 while specificity ranged from 0.95 to 0.98. Analysis of periprosthetic tissue can be valuable for the diagnosis of PJI. Although the histology provides a higher sensitivity than that of tissue culture [5, 20, 21], it does not isolate microorganisms. Tissue culture is a common method for the microbiological diagnosis of PJI. Recently, the new technique of periprosthetic tissue culture in BCB has been used in the clinical setting. The authors in one study found that periprosthetic tissue culture in BCB offered better culture results than traditional medium culture methods. The sensitivity of periprosthetic tissue culture in BCB (87%) is higher than direct solid media (agar plates), cooked meat broth, and fastidious anaerobic broth (39, 83, 57%, respectively) [19]. One reason for the lower sensitivity of the conventional method, may be due to the incubation period of only 5 days of these growth media. Fink and colleagues showed higher sensitivity of tissue culture inform patients with hip and knee PJI (73 and 78%, respectively) when the incubation was prolonged to 14 days [22, 23]. In another study investigating the arthroscopic tissue biopsy in hip PJI, the culture sensitivity was reported even higher (87.5%), along with a specificity of 100% [24]. In a prospective cohort study of 369 participants, the sensitivity and specificity of periprosthetic tissue culture in BCB was compared with those of standard agar (aerobic and anaerobic agar) and thioglycolate broth culture. When using IDSA criteria for diagnosis of PJI, the sensitivity of BCB was higher than that of conventional agar and broth cultures (60.7% vs. 44.4%; *P* = 0.003). However, when Bayesian latent class modelling (LCM) was applied, BCB was associated with a 47% improvement in sensitivity compared with that of conventional agar and broth cultures (92.1% vs. 62.6%). Notably, 51% (60 patients) of patients with PJI had received antibiotic therapy before sample collection, which may influence the sensitivity of all the aforementioned diagnostic tests [1]. However, periprosthetic tissue in BCB improved culture results in patients with previous antibiotic treatment before sample collection. This is because the resin or charcoal present in the BCB could neutralize the effects of antimicrobials, and the ability of resin to neutralize antibiotic activities yields superior results [25]. In the study performed by Yan and co-workers, 27 of 38 patients who received antimicrobial therapy within 4 weeks before surgery demonstrated a positive periprosthetic tissue culture in BCB [13]. In addition, sonication fluid cultures were positive in 29 patients.

Rapid detection of pathogens at an early stage can greatly help the outcome of subsequent treatment. A previous study using an automated BCB system showed that the microorganism could be detected faster than with the conventional method [1]. Minassian et al. showed periprosthetic tissue culture in BCB results in the growth of most microorganisms within 3 days, with aerobic cultures detecting 95% of organisms within 3 days and anaerobic cultures detecting 96% of organisms within 5 days. The authors also say that prolonged microbiological culture for 2 weeks is unnecessary when using BCB [14]. In an additional study, aerobic and anaerobic BCB were positive within the first day of incubation. The aerobic BCB detected pathogen growth more rapidly than any other cultures (anaerobic BCB; thioglycolate broth, and agar), followed by anaerobic BCB. Following 7 days of incubation, no organism was detected in aerobic BCB. The prolonging of anaerobic BCB incubation to 14 days lead to the diagnosis of an additional three PJI as well as the detection of three additional contaminants (i.e. *Cutibacterium acnes*). Although extending the incubation time from 7 to 14 days did not demonstrate any major changes in sensitivity and specificity, the authors support using an incubation approach of 7 days for aerobic BCB and 14 days for anaerobic BCB [1].

Bacterial culture is the key for the diagnosis of PJI, yet culture negative, false positive, or false negative results remain a challenge. The most frequently cultured microorganisms causing PJI are coagulase-negative staphylococci (30–43%), *Staphylococcus aureus* (12–23%), *streptococci* (9–10%), and *enterococci* (3–7%) [26]. *Cutibacterium* spp. is still diagnosed rarely, but requires attention in patients with nonspecific signs or symptoms [27]. Several studies have shown that periprosthetic tissue culture in BCB was able to detect slow-growing organisms such as *Cutibacterium* spp. and was more sensitive and rapid than normal medium. However, false positive cases caused by contamination in this meta-analysis were surprisingly rare. This observation may be explained by careful manipulation in order to minimize contamination [1, 11, 13, 14].

For the intraoperative diagnosis of PJI, the IDSA guideline suggested that ideally five or six periprosthetic tissue samples should be obtained in revision arthroplasty [6]; Peel et al. reported that with the use of the BCB technique, the greatest accuracy of diagnosis could be achieved with only three periprosthetic tissue samples (92%; 95% CI, 79 to 100%) [28]. The culture of periprosthetic tissue in BCB is not only more accurate than conventional culture methods, but also a more cost-effective way to diagnose PJI. One study result demonstrated a 60.1% reduction in mean total staff time with the adoption of periprosthetic tissue culture in BCB compared to conventional techniques (mean ± standard deviation, 30.7 ± 27.6 vs 77.0 ± 35.3 h per month, respectively; *P* < 0.001). The estimated annualized labour cost savings of culture using blood culture bottles was $10,876.83 (± $337.16) [29].

Although periprosthetic tissue in BCB is a valuable method in the diagnosis of PJI, 100% accuracy was not achieved, therefore, a combination of tests can be used to effectively enhance diagnostic accuracy [5]. In a study of periprosthetic tissue from the hip, knee, shoulder, and elbow, combination testing of periprosthetic tissue in BCB with sonicate fluid had the highest sensitivity without compromising specificity. Periprosthetic tissue in BCB was shown to have a similar sensitivity with sonicate fluid (66.4% vs. 73.1%, respectively). In patients with previous antimicrobial therapy, positive cultures of periprosthetic tissue in BCB was 71.1% whereas the percentage of positive cultures in sonicate fluid was 76.3% [13]. Compared with traditional tissue culture, sonication was more sensitive than tissue culture in diagnosing PJI with or without antibiotic treatment [30]. Furthermore, sonication fluid demonstrated comparable effective diagnostic method in patients with infected endoprosthetic reconstructions in treatment of bone tumors, orthopedic hardware (fracture-fixation device or spinal implant) [21, 31], and is also suitable for the diagnosis of PJI in the two-stage revision with antibiotic-loaded cement spacers [32]. Dithiothreitol (DTT) is a strong reducing agent frequently used in microbiology laboratories to liquefy specimens and was suggested for biofilm dislodgement from the implant surface. In a clinical study performed by Sambri and colleagues, the sensitivity of DTT and sonication was found to be similar (91% vs. 89%), with both demonstrating higher sensitivity levels than conventional tissue samples (79%) when using the MSIS criteria as the reference standard for defining PJI [33]. DTT not only detects the microorganism from the implant but is also useful for tissue culture in the diagnosis of bone and joint infections. In a study comparing tissue samples treated with DTT and normal saline solution in cases of orthopedic infection, DTT showed higher sensitivity and specificity than saline (sensitivity: 88.0% vs. 72.0%; specificity: 97.8% vs. 91.1%, respectively) [34]. Another study showed inferior performance of DTT compared to sonication in the diagnosis of biofilm infection [35]. The methods of sonication, DTT and periprosthetic tissue are useful intraoperative methods in the clinical diagnosis of PJI; however, periprosthetic tissue culture does not require additional hardware or devices and is a conventional technique applied in most hospitals. In patients with suspected infection and accompanying joint pain, biopsy and culture without implant removal could be performed under regional anaesthesia at an early stage [36, 37].

There were several potential limitations in this meta-analysis. First, there was no gold standard for diagnosing PJI among the included studies, which had different reference standards. Therefore, the estimates of diagnostic accuracy of a tested method would be underestimated. Second, due to the limited data, we could not conduct subgroup analysis to assess the diagnostic accuracy of periprosthetic tissue BCB in patients with previous antibiotic treatment. Whether the method of periprosthetic tissue BCB improves the result in patients who received antibiotic treatment has not been elucidated to date. Third, periprosthetic tissue culture is one of the most valuable microbiological methods to diagnose PJI. In the present study, only the periprosthetic tissue in BCB was analyzed; due to the limited data of the included studies, a comparison with conventional culture methods was not performed.

## Conclusion

In conclusion, the present study showed that periprosthetic tissue culture in BCB had adequate, clinically acceptable diagnostic values for detecting PJI, with a sensitivity of 70% and a specificity of 97%. Considering the potential limitations, more large-scale, well-performed studies are needed to verify our findings, especially in combination with biofilm removal methods.

## Data Availability

Data was extracted from references [1, 13, 14, 19].

## Abbreviations

AUC: Area under the curve
BCB: Blood culture bottles
CI: Confidence interval
DOR: Diagnostic odds ratio
DTT: Dithiothreitol
EBJIS: European bone and joint infection society
FN: False-negative
FP: False-positive
H: Histological examination
IDSA: Infectious Diseases Society of America
LCM: Latent class modelling
M: Microbiological or laboratory examination
MeSH: Medical subject headings
MSIS: Musculoskeletal Infection Society
NA: Not available
NLR: Negative likelihood ratio
P: Presence sinus tract or purulence around the prosthesis
PJI: Periprosthetic joint infection
PLR: Positive likelihood ratio
SROC: Summary receiver operating characteristic
TN: True-negative
TP: True-positive
